# Fatal Effects Attributed to Chloroform

**Published:** 1851-05

**Authors:** 


### SURGERY.

*Fatal Effects Attributed to Chloroform.*—Dr. Geo. Roe, of Cavan Infirmary, Ireland, records, in the Dublin Med. Press, 26th March, 1851, the following case of death from the use of chloroform, which occurred in September last. The patient was a young man, 24 years of age, suffering from extensive syphilitic ulceration of the foot and leg. Notwithstanding the existence of deep seated tubercles was suspected, at the earnest request of the young man, amputation of the leg was resolved on, which Dr. Roe proceeded to attempt, he informs us, "on the 20th of September, having promised I would give him something to

*numb and take away the pain* of the operation, as he said I had done to others.

He was of a very peculiar and very sensitive temperament, yet very patient and uncomplaining, and I knew he endeavored to conceal feelings and expressions which many with less cause or suffering would give way to. For this reason, I wished by every means to allay and prevent the agitation and excitement inseparable from the operation, by telling him I would perform it in the next room, and not require him to be carried down stairs to the operation room ; I also placed the tourniquet on his thigh while in his bed, previous to his removal to the table.

He was cheerful and appeared to be firm and courageous, but when placed on the table, the heart's action was very *quick and weak,* but he did not appear faintish, or more pale than usual. I *then saw* Mr. Nalty, the apothecary, *measure one drachm* of the chloroform, in the small minim glass measure, and pour it on a little folded lint, which was placed in an oval hollowed sponge, held in the hand with a small towel. Recollecting I had used this chloroform in another case, and finding some little delay in producing the anæsthetic effects, and supposing the strength of the chloroform might be a little weakened, as the bottle had not been kept very closely stopped, I directed Mr. Nalty to add *thirty* drops more to that already put on the lint.

I then applied the sponge, &c., to the patient's nose, directing him to keep his mouth shut ; I then gave the towel, &c., to the care of Dr. Halpin, who was at the opposite side of the table, while I went to prepare myself for the operation. Mr. Brice had scarcely screwed the tourniquet, which had been placed previously on the thigh, and while I was examining the state of the circulation in the tibial arteries, to prevent the least unnecessary loss of blood, which could not have occupied one minute—certainly he could not have made or taken *fifteen* inspirations,—when Dr. Halpin told me the anæsthetic effects were produced. This struck me as being unusually quick and sudden, and on removing the towel from the face we saw a slight convulsive action of the left eyelids, and the lids partially open, and a small quantity of saliva (frothy) at the mouth. I felt rather uneasy, but not much alarmed, as Dr. Halpin said he had often seen such symptoms from the effect of chloroform, but which I had not met with ; and on a more minute and instant examination of the heart, the eyes, muscles of the limbs, &c., we found him *dead.* Every means within our reach were resorted to, to try and restore animation. The strongest ammonia and hartshorn were applied to the nostrils, the fauces and palate ; cold air, cold water, to to the surface ; and afterwards scalding water over the region of the heart ; inflation of the lungs ; general friction of the body ; change of posture, were all in turn and rapid succession, tried, but without the least effect. I lament I had no means of making or procuring oxygen gas, and unfortunately my little portable galvanic apparatus was not then in order or ready for use ; so that we had the sad and painful spectacle of our patient *killed*, as if by a stroke of lightning, in less than *one* minute, before our eyes."

